# Photopsias during Systemic Bevacizumab Therapy

**DOI:** 10.1155/2016/1926178

**Published:** 2016-03-16

**Authors:** Heather Leisy, Meleha Ahmad, R. Theodore Smith

**Affiliations:** Department of Ophthalmology, New York University School of Medicine, New York, NY 10016, USA

## Abstract

*Background.* The authors describe a case of recurrent photopsias in a 56-year-old woman following repeat treatments with systemic intravenous bevacizumab for stage IV ovarian cancer. To our knowledge, this is the first report of photopsias following systemic bevacizumab treatments in a patient with a normal eye exam.* Case Presentation.* A 56-year-old Caucasian female complained of onset of floaters and flashes in the temporal peripheral field of the right eye 1 day after receiving intravenous of 30 g of 25 mg/mL of systemic bevacizumab for treatment of stage IV ovarian cancer. Ophthalmic examination, including dilated fundus exam, spectral domain optical coherence tomography (SD-OCT) of the optic nerve head, and enhanced depth imaging SD-OCT of the macula, revealed no significant abnormalities. Possible mechanisms are reviewed.* Conclusion.* We propose that patients who undergo intravenous bevacizumab treatments are questioned for any ocular symptoms and that more systematic evaluations of retinal nerve fiber layer and choroidal effects are obtained in those patients who are on long-term treatment at high doses.

## 1. Background

Bevacizumab is a humanized monoclonal immunoglobulin G antibody that exerts an antiangiogenic effect by binding to any isomer of vascular endothelial growth factor A (VEGF-A) [1]. Originally approved in 2004 to treat metastatic colorectal cancer [2], it quickly found ophthalmic applications in the treatment of neovascular age-related macular degeneration (AMD), first as systemic therapy [3] and later intravitreally [4]. The only previously reported ocular side effects from systemic bevacizumab therapy—used in ophthalmologic or oncologic settings—are mild epiphora and optic nerve dysfunction [3–7]. Intravitreal bevacizumab injection has been associated with a number of adverse effects, including intraocular inflammation or infection, retinal pigment epithelium (RPE) tear, retinal detachment, and vitreous hemorrhage [8]. The reasons for these events remain unclear; mechanical and drug-related causes have been hypothesized [8, 9]. Here, we describe the first reported case of a patient with no ocular history who experienced photopsias while undergoing chemotherapeutic treatment with systemic bevacizumab and review possible mechanisms.

## 2. Case Report

**Figure 1:**
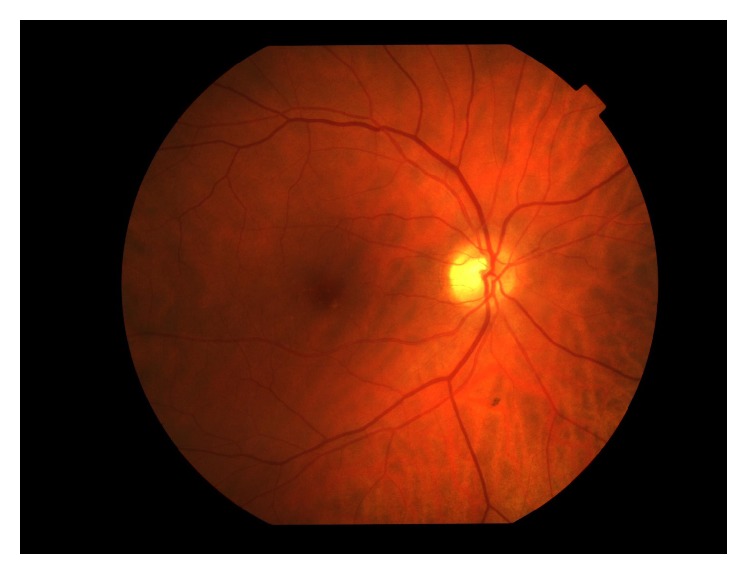
Color fundus photography of right eye with rare macular hard drusen and scattered peripheral retinal pigment.

**Figure 2:**
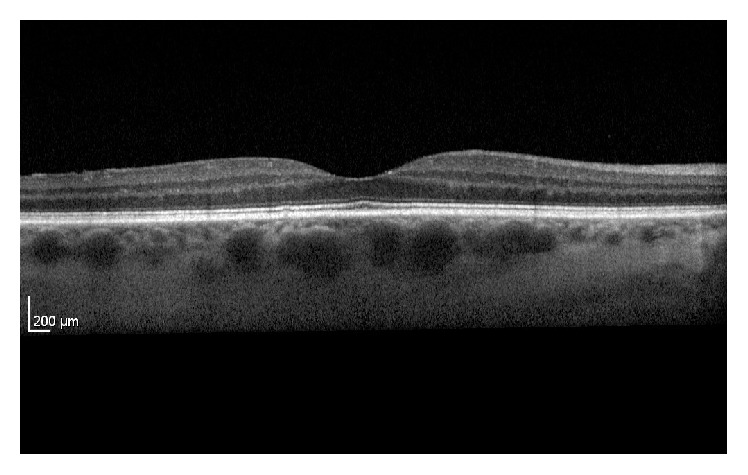
Enhanced depth imaging optical coherence tomography image of right eye showing normal macular anatomy with choroidal thickness of 340 *µ*m.

A 56-year-old woman with stage IV ovarian cancer undergoing maintenance treatment with systemic bevacizumab presented to ophthalmology clinic complaining of 6 days of flashes and floaters in her right eye. Her symptoms began suddenly 1 day after her second cycle of bevacizumab given alone by intravenous infusion (30 g at 25 mg/mL for 1 hour). Previously, she had received 6 consecutive cycles of paclitaxel and bevacizumab in combination but was switched to bevacizumab alone due to neuropathy. Photopsias were described as a “shooting planet” with a broad “tail” occurring several times in a row in the temporal peripheral field of the right eye, with episodes spaced sporadically throughout the day. She was relatively symptom free at the time of presentation, with the exception of a rare streak of light with movement and a few mild floaters. She had no ophthalmic history or associated symptoms such as headache, photophobia, blurred vision, or neuropathy. Best-corrected visual acuity was 20/20-2 OD and 20/20-2 OS. Anterior segment exam, intraocular pressure, pupillary light reflexes, color vision, visual field testing, and dilated fundus exam were normal except for rare macular hard drusen and scattered peripheral retinal pigment (Figure 1). Spectral domain optical coherence tomography (SD-OCT) of the retina with and without enhanced depth imaging (EDI) revealed no evidence of posterior vitreous detachment (PVD) or other abnormalities (Figure 2). SD-OCT of the optic nerve was within normal limits. Electroretinogram (ERG) could not be obtained due to difficulties in coordinating her existing chemotherapy schedule with the scheduled times of ERG availability at our institution. Given low clinical suspicion for vascular leakage, angiography was deferred. Upon follow-up, she reported the same symptoms occurring after 2 subsequent treatments of bevacizumab alone spaced 3 weeks apart. In both occurrences, symptoms appeared 1 day after treatment and lasted for approximately 1 week. Her eye exam remained unchanged at follow-up visits. Standard laboratory findings, including basic metabolic panel, complete blood count, and lipid panel, were within normal limits.

## 3. Conclusion

To our knowledge, this is the first documented case of photopsias occurring in a patient being treated with systemic intravenous bevacizumab. Based on the WHO-UMC causality classification system, causality is probable to certain (time relationship plausibility, disease unrelated, withdrawal response, and satisfactory rechallenge) [10]. Possible mechanisms for the sudden development of photopsias following a dose of systemic bevacizumab are neuronal, vascular, structural, and inflammatory. A dose-dependent effect may explain the onset of symptoms following 7 completed cycles of treatment. This is consistent with other dose-dependent side effects of intravenous bevacizumab such as hypertension and proteinuria [11], bleeding [12], and impaired wound healing [13].

*Neuronal.* VEGF is expressed in RPE cells, Müller cells, and the vascular endothelial cells of the retina where it influences retinal neuronal development, growth, and stability [14, 15], giving anti-VEGF the potential to affect neuronal function upon crossing the blood retinal barrier (BRB). Although the retina is typically thought of as immune privileged, animal studies have shown that bevacizumab may, in fact, be able to cross the BRB even in nondisease states [16, 17]. In our patient's case, high baseline VEGF levels due to ovarian cancer [18] may result in leakiness of this barrier [19], allowing bevacizumab accumulation in the retina. Neuronal death and subsequent photopsias thus may develop through a bevacizumab-induced decline in VEGF levels. Systemic bevacizumab, administered at dosing intervals of 2 or 3 weeks, has been shown to significantly lower serum-free VEGF levels [20], and VEGF neutralization led to an observed increase in apoptosis of neuronal cells in the inner and outer nuclear layers in mice [21]. A series of 6 cases of optic neuropathy developing after systemic bevacizumab treatment for glioblastoma was reported [7], and this effect may have been due to the loss of VEGF's known neuroprotective effects with subsequent neuronal degeneration [14]. Of note, these patients had received a mean of 7.5 doses of bevacizumab prior to the onset of visual symptoms and half were noted to have a normal optic apparatus on MRI imaging [7]. Retinal degeneration has been known to cause photopsias in other disease processes such as retinitis pigmentosa and Best disease, presumably due to aberrant photoreceptor stimulation [22].

*Vascular.* Healthy RPE secretes VEGF at its basolateral side to maintain the choriocapillaris [23]. Decreased secretion of VEGF by the RPE, causing an ischemic effect on the choroid and outer retina, presents one mechanism by which systemic bevacizumab could cause photopsias. Intravitreal bevacizumab has been shown to significantly reduce choriocapillaris endothelial cell fenestration and to promote thrombosis through leukocyte plugging and thrombocyte activation in primate eyes [24]. Clinically, these injections are associated with both systemic [25] and ocular [26] ischemic events. Systemic treatment has been linked to vascular endothelial dysfunction, decreased vasodilatory response [27], and cerebral and cardiac ischemic events [28]. This treatment might be similarly associated with ocular ischemia, although not yet reported in the literature [4]. While there is no evidence for overt ocular vascular damage in our patient, transitory ischemia remains a possibility.

*Structural.* In this case, no appreciable vitreous detachment was observable on either clinical exam or SD-OCT. However, subclinical vitreous destabilization or disturbance of the vitreoretinal interface outside of the imaging field remains a possibility. Other systemically administered chemotherapeutic agents have been associated with posterior vitreous detachment [29].

*Inflammatory.* Bevacizumab has been hypothesized to be more immunogenic or proinflammatory due to its larger Fc portion and a longer half-life compared with other anti-VEGF agents, and it has been shown to induce the expression of inflammatory cytokines when injected intravitreally [30]. Systemic bevacizumab infusion has been linked to a single case of optic neuritis in a patient undergoing treatment for metastatic melanoma [6], and intravitreal use has been linked to cases of uveitis [31]. In our patient, there were no clinical signs of intraocular inflammation, but this does not preclude subclinical inflammation of photoreceptors causing photopsias.

Alternative nonocular causes of photopsias, such as ophthalmic migraine, hypoglycemia, vertebrobasilar insufficiency, metastatic adenocarcinoma of the central nervous system, and severe cough, must also be considered as unusual but possible causes for photopsias [22]. However, these etiologies are all more likely to cause central or bilateral photopsias and to present with other systemic symptoms. Functional side effects of chemotherapeutic agents should be considered in patients presenting with ocular symptoms while undergoing chemotherapy.

## Abbreviations

SD-OCT: Spectral domain optical coherence tomography
VEGF-A: Vascular endothelial growth factor A
AMD: Age-related macular degeneration
RPE: Retinal pigment epithelium
EDI: Enhanced depth imaging
ERG: Electroretinogram
BRB: Blood retinal barrier.

## Additional Points

Data and materials are available at Department of Ophthalmology, New York University School of Medicine, New York, NY.
